# The awareness and practice of dentists regarding medication-related osteonecrosis of the jaw and its prevention: a cross-sectional survey

**DOI:** 10.1186/s12903-021-01475-6

**Published:** 2021-03-24

**Authors:** A. Lum Han

**Affiliations:** Department of Family Medicine, Wonkwang University Hospital, Iksan, Jeollabuk-do 54538 South Korea

**Keywords:** Bisphosphonate-associated osteonecrosis of the jaw, Awareness, Dentists, Education, Osteoporosis

## Abstract

**Background:**

Accurate documentation of a patient’s prior medication use and awareness of side effects associated with anti-osteoporotic agents can assist dentists to prevent medication-related osteonecrosis of the jaw. I aimed to determine the awareness of Korean dentists regarding medication-related osteonecrosis of the jaw and the duration of drug holidays they prescribe to patients who need to undergo various dental procedures.

**Methods:**

An online, questionnaire-based survey was conducted among 1000 dentists registered in an online community in Korea. The following were determined: general characteristics; type of practice; recordkeeping regarding patients’ use of bone-modifying agents; requirement of a doctor’s referral letter; advice given regarding drug holidays of bone-modifying agents before dental surgery procedures; and experience with medication-related osteonecrosis of the jaw. Differences between dentists with and without experience in treating patients with medication-related osteonecrosis of the jaw were evaluated using the χ^2^ test.

**Results:**

Although a relatively high proportion (293/1000, 29.3%) of dentists had experienced cases of medication-related osteonecrosis of the jaw, only 650/1000 (65.0%) routinely documented the type of bone-modifying agent used by patients and the duration of its use. Moreover, only 591/1000 (59.1%) dentists routinely requested referral letters from doctors before performing dental surgery on patients. Although the recommended period for a drug holiday differs for each drug, 533/1000 (53.3%) dentists did not make such a distinction. There was a statistically significant difference in the level of detail documented in terms of anti-osteoporotic drug use between dentists who had no experience in medication-related osteonecrosis of the jaw (707/1000) and those who had such experience (*P* = 0.007). There was a statistically significant difference in the length of drug holidays prescribed between dentists with and without prior experience with the condition (*P* = 0.001).

**Conclusions:**

These results suggest that dentists do not respond consistently to patients' drug history prior to performing dental procedures. This implies the need for increased cooperation between dentists and physicians, as well as the development of targeted educational interventions for the dental profession, to reduce the risk of medication-related osteonecrosis of the jaw.

**Trial registration:**

Not applicable.

**Supplementary information:**

The online version contains supplementary material available at 10.1186/s12903-021-01475-6.

## Background

Several medications decelerate or prevent bone loss. However, patients on bone-modifying medications should be treated with caution by dentists because of the possibility of medication-related osteonecrosis of the jaw (MRONJ). Many studies on MRONJ have been conducted since the first report by Marx (2003) [1] almost two decades ago. In 2014, the American Association of Oral and Maxillofacial Surgeons revised their recommendations on diagnosis and treatment strategies for the condition [2]. One of the updated recommendations was to replace the term “bisphosphonate-related osteonecrosis of the jaw” (BRONJ) with “MRONJ.” This was done following many reports of MRONJ that were associated with the use of bone-resorption inhibitors, such as denosumab (e.g., Prolia and Xgeva [Amgen Inc., Thousand Oaks, CA, USA]), and angiogenesis inhibitors, since the publication of their first position paper [2].

In the United States, the prevalence of BRONJ in patients on intravenous bisphosphonate therapy has been reported as 0.7/100,000 (0.8%–12%) [3]. Using a survey, the prevalence of BRONJ in Europe has been reported as 95/100,000 (0.095%) and 1/100,000 (0.001%) for patients receiving intravenous and oral administration, respectively [4]. In another study, a low prevalence of BRONJ (0.004%) was identified among patients on oral therapy than among those on intravenous therapy [5]. Lo et al. (2010), on the other hand, documented an increase in prevalence of BRONJ from 0.04 to 0.21% after more than 4 years of continued bisphosphonate therapy [6]. The prevalence of MRONJ in patients receiving denosumab was 0.085% in a study in which changes were monitored in women with postmenopausal osteoporosis after 5 years’ exposure to denosumab [7]. Therefore, although the prevalence of MRONJ varies across countries, its general prevalence appears to be relatively low.

There are several risk factors for MRONJ. These include the patient's underlying disease, duration of administration of anti-osteoporosis drugs, whether or not to extract teeth, its anatomical structure, whether to use dentures, and accompanying oral diseases. [8, 9].

Due to the long skeletal retention time of bisphosphonates, its inhibitory effect on bone resorption remains for a time after its discontinuation. Moreover, the long-term use of bisphosphonates causes side effects such as BRONJ and atypical femoral fractures. To prevent such side effects, physicians normally propose a "drug holiday." Patients at low risk of fracture are recommended to take a drug holiday after taking oral or intravenous bisphosphonates for 5 or 3 years, respectively. Because the effects of denosumab do not persist after treatment is discontinued, a drug holiday for patients taking this drug is not necessary [7, 10].

Dentists have an important role in assessing the risk of MRONJ by accurately documenting their patients’ medical and drug history. An inadequate knowledge of this medical condition and the option to discontinue bone-modifying medications (i.e., to take a drug holiday) in patients with osteoporosis can have serious consequences. Therefore, the awareness and perceptions of dentists regarding MRONJ should be analyzed in order to identify existing knowledge deficiencies and inform the development of targeted educational interventions in the dental profession.

Studies in this field are scarce and the level of awareness among dentists likely varies across countries due to differences in training and definitive treatment protocols. Thus, I investigated the awareness of Korean dentists regarding MRONJ, as well as the durations of drug holidays they advised their patients to take before undergoing various dental procedures. The overarching aim with this study was to determine the need for cooperation between dentists and physicians in terms of MRONJ, as well as for enhanced educational protocols for the dental profession.

## Methods

### Ethical approval

This study was approved by the institutional bioethics committee of our university hospital (approval number: 2019-03-015) and conducted in accordance with the principles of the Declaration of Helsinki. Informed consent was obtained from all study participants.

### Participants

According to health insurance statistics of 2017, approximately 25,300 dentists practice in Korea. This study was conducted among dentists registered on DentPhoto (http://www.dentphoto.com/), the largest online community of dentists in Korea.

### Survey method

The survey was initiated online in April 2020 and closed automatically after it was completed by 1000 dentists. I prepared the questionnaire by adapting existing research data [11]. I developed the questionnaire by myself as part of the study. I used it to record general characteristics of the dentists, such as sex, age, clinical experience, and type of practice. The type of practice was categorized as either private (i.e., dentists engaged in private practice) or non-private (e.g., employed dentists and public dentists). Questions were also included to determine the following: whether dentists recorded details of current or prior usage of bone-modifying agents (e.g., the drug name and duration of therapy) before implant placement or tooth extraction; the required period of drug discontinuation before treating patients; specific management protocols employed while treating patients on bone-modifying medications; and prior experience with MRONJ.

### Statistical analysis

IBM SPSS Statistics for Windows version 21.0 (IBM Corp., Armonk, NY, USA) was used for data analysis. Frequencies were calculated and cross-tabulation analyses were performed to evaluate knowledge pertaining to MRONJ among dentists. Data were presented as frequencies (%), as all variables were categorical. Differences between dentists with and those without prior experience in treating patients with MRONJ were evaluated using the χ^2^ test. The level of statistical significance was set at *P* < 0.05.

## Results

### General characteristics of survey respondents

The majority of survey respondents were male (78.7%) and almost half (46.6%) were 41–50 years of age. Dentists engaged in private practice accounted for 85.3% of survey respondents, with the remainder being engaged in non-private practice. There was wide variation in clinical experience in dental practice (Table 1).

**Table 1:** General characteristics of survey respondents

Variable	N	%
*Sex*		
Male	787	78.7
Female	213	21.3
*Age (y)*		
≤ 30	32	3.2
31–40	298	29.8
41–50	466	46.6
51–60	172	17.2
≥ 61	32	3.2
*Type of practice*		
Private clinic^a^	853	85.3
Non-private clinic^b^	147	14.7
*Clinical experience (y)*		
≤ 5	102	10.2
6–10	179	17.9
11–15	256	25.6
16–20	212	21.2
≥ 21	251	25.1

y, year

^a^Dentists engaged in private practice

^b^Employed dentists, public dentists, etc.

### Dentist protocols, awareness of MRONJ, and drug holidays

Most (96.9%) of the dentists reported that they regularly documented the medication history (including that of bone-modifying agents) of patients before implant placement or tooth extraction. Over half (65.0%) recorded both the drug name and duration of use, while 16.3% of the respondents only documented the drug name. Referral letters were requested by 59.1% of respondents prior to dental surgery procedures. In terms of the time that patients were advised to discontinue anti-osteoporotic agents before tooth extraction or implant placement, 45.7% of respondents indicated that it was 3–5 months; 14.9% and 39.4% of respondents indicated that it was ≤ 2 and ≥ 6 months, respectively (Table 2).

**Table 2:** Dentists’ awareness of MRONJ and protocol followed before dental procedures

Variable	N	%
*Do you record if the patient is taking anti-osteoporotic drugs?*		
Yes	969	96.9
No	31	3.1
*Do you record the name of the drug and duration of its use?*		
No	187	18.7
Name only	163	16.3
Name and duration	650	65.0
*Do you ask for a doctor's referral letter prior to dental surgery procedures?*		
Yes	591	59.1
No	409	40.9
*How long do you advise patients to discontinue the anti-osteoporotic agent before tooth extraction or implant placement?*		
≤ 2 months	149	14.9
3–5 months	457	45.7
≥ 6 months	394	39.4
*Do you recommend different drug holidays depending on the type of drug (bisphosphonates, denosumab, SERMs, PTH)?*		
Yes	467	46.7
No	533	53.3
*If you have encountered case/s of MRONJ, what type of medication caused it?*		
Bisphosphonates	276	27.6
Denosumab	13	1.3
SERMs	4	0.4
No experience	707	70.7

MRONJ, medication-related osteonecrosis of the jaw; SERMs, selective estrogen-receptor modulators; PTH, parathyroid hormone

Different durations of drug holidays, depending on the type of bone-modifying agent (bisphosphonates, denosumab, selective estrogen-receptor modulators, or parathyroid hormone) used, were advised by 46.7% of respondents. MRONJ was most often reported to result from bisphosphonate therapy (27.6%); denosumab therapy was cited as the cause of MRONJ by only 1.3% of respondents. Almost three-quarters (70.7%) of respondents reported no prior experience of treating patients with MRONJ (Table 2).

### Awareness and specific management according to prior MRONJ experience

The largest proportion (689/969, 71.1%) of dentists who documented anti-osteoporotic medication use had no prior experience with MRONJ (Table 3). There was a statistically significant difference in the level of detail documented in terms of anti-osteoporotic drug use between dentists who had no experience in MRONJ and those who had (*P* = 0.007). There was a statistically significant difference in the length of drug holidays prescribed between dentists with and those without prior experience of MRONJ (*P* = 0.001). There was no significant difference in the proportion of dentists who recommended different periods of drug holidays (depending on drug type) between those with and those without prior experience with MRONJ.

**Table 3:** Awareness and management protocol according to prior MRONJ experience

Sex	MRONJ experience		*P*^a^
	No	Yes	
Male	541 (76.5%)	246 (84.0%)	0.009
Female	166 (23.5%)	47 (16.0%)	
*Age (y)*			
≤ 30	5 (0.7%)	27 (9.2%)	< 0.0001
31–40	226 (32%)	72 (24.6%)	
41–50	350 (49.5%)	116 (39.6%)	
51–60	102 (14.4%)	70 (23.9%)	
≥ 61	24 (3.4%)	8 (2.7%)	
*Type of practice*			
Private clinic^b^	605 (85.6%)	248 (84.6%)	0.705
Non-private clinic^c^	102 (14.4%)	45 (15.4%)	
*Clinical experience (y)*			
≤ 5	64 (9.1%)	38 (13%)	0.030
6–10	135 (19.1%)	44 (15%)	
11–15	193 (27.3%)	63 (21.5%)	
16–20	150 (21.2%)	62 (21.2%)	
≥ 21	165 (23.3%)	86 (29.4%)	
*Do you record if the patient is taking anti-osteoporotic drugs?*			
Yes	689 (97.5%)	280 (95.6%)	0.116
No	18 (2.5%)	13 (4.4%)	
*Do you record the name of the drug and duration of its use?*			
No	115 (16.3%)	72 (24.6%)	0.007
Name only	115 (16.3%)	48 (16.4%)	
Name and duration	477 (67.5%)	173 (59.0%)	
*Do you ask for a doctor's referral letter prior to dental surgery procedures?*			
Yes	423 (59.8%)	168 (57.3%)	0.466
No	284 (40.2%)	125 (42.7%)	
*How long do you advise patients to discontinue the anti-osteoporotic agent before tooth extraction or implant placement?*			
≤ 2 months	88 (12.4%)	61 (20.8%)	0.001
3–5 months	321 (45.4%)	136 (46.4%)	
≥ 6 months	298 (42.1%)	96 (32.8%)	
*Do you recommend different drug holidays depending on the type of drug (bisphosphonates, denosumab, SERMs, PTH)?*			
Yes	319 (45.1%)	148 (50.5%)	0.120
No	388 (54.9%)	145 (49.5%)	
*If you have encountered case/s of MRONJ, what type of medication caused it?*			
Bisphosphonates	–	276 (94.2%)	–
Denosumab	–	13 (4.4%)	
SERMs	–	4 (1.4%)	
No experience	707 (100.0%)	–	

y, year; MRONJ, medication-related osteonecrosis of the jaw; SERMs, selective estrogen-receptor modulators; PTH, parathyroid hormone

^a^Differences between dentists with and those without prior MRONJ experience were evaluated using the χ^2^ test. The level of statistical significance was set at *P* < 0.05. Data are presented as N (%)

^b^Dentists engaged in private practice

^c^Employed dentists, public dentists, leave of absence, etc.

## Discussion

In this study, most dentists (65.0%) recorded both the name of the anti-osteoporotic drug and its duration of use. While the recommended period for a drug holiday differs for each drug, most (53.3%) dentists did not make such a distinction.

There were no statistically significant differences in whether bone-modifying medications were recorded or whether a referral from the attending physician was requested prior to dental surgery procedures between dentists with and those without experience with MRONJ. This result suggests what are necessary for the prevention of MRONJ. In a previous study, it was reported that the proportion of dentists who did not contact or ask for referral from an attending physician prior to dental procedures was low, and that it decreased slightly from 2014 (12.86%) to 2018 (11.67%) [11]. The authors of that study noted that such unsupervised procedures can reduce patients' quality of life.

We observed that more dentists advised patients to take drug holidays ≥ 6 months than ≤ 2 months for the prevention of MRONJ. This period is longer than that recommended in the literature [7, 10]. Hence, the optimal duration of a drug holiday cannot be determined from the results of our study alone, and further long-term studies are required for definitive conclusions to be drawn.

Due to the aging population, not only in Korea but also worldwide, an increasing number of elderly individuals with multiple co-morbidities are expected to undergo dental procedures, including implant placement. Thus, an awareness of the potential interactions in treatment regimens prescribed to the same patient by doctors in different fields of specialization will become increasingly important, necessitating optimal communication between healthcare professionals.

Poor patient compliance is one of the primary obstacles in the treatment of osteoporosis, commonly resulting from side effects of bone-modifying agents, or the fear thereof [12, 13]. On the other hand, excessive use of bone-modifying agents can increase the risk of MRONJ. MRONJ is a rare but serious complication following treatment with certain medications, and is defined as the presence of exposed bone in the oral and maxillofacial regions (or extra- or intra-oral fistulas) lasting more than 8 weeks. To be diagnosed with MRONJ, a patient should have no prior history of radiation, or of treatment with bone-resorption inhibitors or angiogenesis inhibitors, for tumor metastasis to the jawbones [7].

The exact mechanism of development of MRONJ is unclear, in spite of several proposed hypotheses. Additionally, varied opinions regarding the efficacy of surgical and non-surgical treatments have been presented [14, 15]. In addition to bisphosphonates, several drugs such as denosumab, steroids, and other angiogenesis inhibitors can cause MRONJ [7, 16]. Denosumab is an anti-human receptor activator of the nuclear factor kappa-Β ligand and inhibits the activity of osteoclasts. It is used to treat osteoporosis caused by bone absorption disorders [17], similar to bisphosphonates, and its cost-effectiveness [18] and convenience of administration has recently led to its increased use in Korea. Denosumab is also associated with a risk for MRONJ; however, the risk of fracture may increase either temporarily or permanently when its use is discontinued [7].

The determination of the type of bone-modifying medication and its duration of use, potential alternative medications, and a drug holiday of 2–3 months before performing dental procedures is essential for preventing MRONJ [7, 10]. The disadvantages of drug discontinuation should be weighed against its advantages. In a post-hoc analysis, Anagnostis et al. (2017) [19] concluded that drug holidays are advisable for patients who have not experienced recent fractures and for those at low risk of fracture, defined as follows: a femoral neck T-score ≥ − 2.5; age < 70 years; and no diseases or medications that could increase fracture risk. Drug holidays should be considered for patients with a 5-year history of alendronate use or a 3-year history of zoledronic acid (or risedronate) use [19, 20]. The duration of the drug holiday should also be based on bone mineral density [20]. The management of the osteoporotic condition with other bone-modifying medications is advisable if denosumab is discontinued [21, 22]. However, drug holidays are not recommended for bone-modifying medications such as denosumab, hormone replacement therapy, selective estrogen-receptor modulators, and teriparatide [21], or for patients with severe osteoporosis [10, 22]. Therefore, the determination of a drug discontinuation protocol may be beyond the scope of dentistry and it is important that dentists request a doctor's referral letter prior to any dental procedure in patients at risk for MRONJ. In addition, detailed records of bone-modifying agents must be requested and maintained. However, the results of our study showed that the proportion of dentists requesting referral letters was relatively low (59.1%). In addition, the proportion of dentists that encountered cases of MRONJ (29.3%) was higher than the general prevalence of MRONJ. This highlights the importance of dentists possessing adequate knowledge pertaining to this condition, and of proper dental management protocols.

Several prior studies have been conducted to examine the awareness of dentists regarding MRONJ in different countries. In a survey of 120 dentists in Romania, the majority were aware of bisphosphonate therapy and its complications, but were not familiar with the pathophysiology, diagnosis, and treatment of BRONJ [23]. In a survey of 204 Brazilian dentists and dental students, researchers discovered a lack of knowledge regarding bisphosphonates and BRONJ [24]. In a survey of 60 dentists and 60 dental students in Spain, 30 (50%) students and 41 (68.36%) dentists were determined to have up-to-date knowledge regarding BRONJ [25]. In a survey of 129 British dentists, more than 90% admitted a lack of awareness regarding drugs (other than bisphosphonates) that cause MRONJ. Furthermore, the lack of a standardized protocol was reported as the primary reason for reluctance in managing such patients [26]. In a survey of 222 Saudi physicians and dentists, only 31.5% were aware of BRONJ. The authors suggested that this could be improved through education [27]. In the present study, we found that the level of experience among dentists regarding MRONJ was high (29.3%); nevertheless, the documentation of patients’ history pertaining to the type of bone-modifying agent and the duration of its use was insufficient (65.0%). Hence, dentists should be made aware of the guidelines for treatment of such patients through regular educational programs.

Our study had some limitations. First, only 1000 dentists were surveyed; therefore, the results may not be generalizable to all dentists in Korea. Second, due to the limited scope of the questions in the survey, it was not possible to elucidate dentists’ knowledge of MRONJ pathogenesis. Third, due to the questionnaire survey system, the number of questions was limited, so detailed surveys were not possible. Nevertheless, the results of this survey can serve as a basis for larger, more detailed, long-term studies to investigate the dentist's perception.

## Conclusions

Elderly individuals often have multiple co-morbidities that require multidisciplinary management. Due to an aging demographic, dentists are likely to encounter an increasing number of elderly patients with osteoporosis who require implant surgery. Thus, the successful management of such patients will require an approach that encourages cooperation between doctors and dentists, as well as the development of educational programs to increase knowledge and awareness of MRONJ.

## Supplementary Information

**Additional file 1:** Supplemental appendices.

## Data Availability

The data will not be shared because it is held by the internet community site of dentists (http://www.akermedia.co.kr/).

## Abbreviations

BRONJ: Bisphosphonate-related osteonecrosis of the jaws
MRONJ: Medication-related osteonecrosis of the jaws
PTH: Parathyroid hormone
SERM: Selective estrogen-receptor modulators
