# Supplementary material for: The awareness and practice of dentists regarding medication-related osteonecrosis of the jaw and its prevention: a cross-sectional survey
Source: BMC Oral Health. 2021 Mar 24;21:155. doi: 10.1186/s12903-021-01475-6 (PMC7992948; doi:10.1186/s12903-021-01475-6)
Supplement: Supplementary file 1 — Additional file 1: Supplemental appendices. [file 12903_2021_1475_MOESM1_ESM.docx]

**Research Topic**: **The awareness and perceptions of dentists regarding medication-related osteonecrosis of the jaw and its prevention: a cross-sectional survey**

**Purpose of the questionnaire**: The accurate documentation of a patient’s prior medication use by dentists, and their knowledge of the side effects associated with anti-osteoporotic agents can prevent medication-related osteonecrosis of the jaws. This study investigated the awareness of dentists regarding medication-related osteonecrosis of the jaws, with the aim of determining the need for targeted educational interventions for this medical condition within the dental profession.

WeIt is for presenting.

발표 용입니다.

This is to suggest.

이것은 제안하는 것입니다.

전체 결과를 로드할 수 없음

다시 시도

재시도 중...

재시도 중...

will not use it for anything other than research purposes.

I will not use it for anything other than research purposes.

연구 목적 외에는 사용하지 않겠습니다.

Except for research purposes is not used.

연구 목적을 제외하고는 사용하지 않습니다.

전체 결과를 로드할 수 없음

다시 시도

재시도 중...

재시도 중...

전체 결과를 로드할 수 없음

다시 시도

재시도 중...

재시도 중...

If you agree to the collection of information, please submit a response questionnaire.

**Questionnaire Provider**: A Lum Han from Wonkwang University Hospital Department of Family Medicine

1. How is the hospital open?

1) Opening of the hospital 2) Non-opening doctors (service doctor, pay doctor, public health doctor, leave of work, etc.)

2. What is your gender?

1) Male 2) Female

3. What is your age range?

1) Under 30

2) 31-40 years old

3) 41-50 years old

4) 51-60 years old

5) 61 years old or older

4. What is the elapsed period after obtaining a dentist's license?

1) 1st year-5th year

2) 6 years-10 years

3) 11 years-15 years

4) 16 years-20 years

5) Over 21 years

5. Do you check whether the patient is taking injections or drugs for osteoporosis treatment at the time of implant or tooth extraction?

1) Yes 2) No

6. Do you identify and record the name and duration of the patient's osteoporosis treatment injection or medication when an implant or tooth is extracted?

1) Drug name record 2) Dosing period record 3) Do not record

5. Do you identify and record the name of the patient's osteoporosis treatment injection or medication taken at the time of implant or tooth extraction?

1) Yes 2) No

6. Do you identify and record the duration of the patient's osteoporosis treatment injections or medication administration during implants or tooth extraction? 1) Yes 2) No

7. Do you request a written statement from the doctor prescribing the osteoporosis drug to the patient?

1) Yes 2) No

8. If surgery is absolutely necessary, how long do you leave osteoporosis medications for at least?

1) Less than 3 months 2) More than 3 months and less than 6 months 3) More than 6 months

9. Do you deal differently with bisphosphonate, denosumab (prolia), SERM (raloxifene, bazedoxifen), and parathyroid hormone drugs?

1) Yes 2) No

10. If you have ever experienced jaw bone necrosis, what medications were you using?

1) Bisphosphonate 2) Denosumab (Prolia) 3) SERM (raloxifene, bazedoxifen)
